# Effect of CXCL12/CXCR4 signaling on neuropathic pain after chronic compression of dorsal root ganglion

**DOI:** 10.1038/s41598-017-05954-1

**Published:** 2017-07-18

**Authors:** Yang Yu, Xini Huang, Yuwei Di, Lintao Qu, Ni Fan

**Affiliations:** 1 The Affiliated Brain Hospital of Guangzhou Medical University (Guangzhou Huiai Hospital), 36 Mingxin Road, Liwan District, Guangzhou, Guangdong Province 510370 China; 2 Department of Pathology and Laboratory Medicine, Guangdong General Hospital, Guangdong Academy of Medical Sciences, Guangzhou, 510080 China; 3 Department of Neurosurgery, Neurosurgery Pain Research Institute, Johns Hopkins University School of Medicine, 725N. Wolfe St., Baltimore, MD 21205 USA

## Abstract

Neuropathic pain is a complex, chronic pain state that often accompanies tissue damage, inflammation or injury of the nervous system. However the underlying molecular mechanisms still remain unclear. Here, we showed that CXCL12 and CXCR4 were upregulated in the dorsal root ganglion (DRG) after chronic compression of DRG (CCD), and some CXCR4 immunopositive neurons were also immunopositive for the nociceptive neuronal markers IB4, TRPV1, CGRP, and substance P. The incidence and amplitude of CXCL12-induced Ca^2+^ response in primary sensory neurons from CCD mice was significantly increased compared to those from control animals. CXCL12 depolarized the resting membrane potential, decreased the rheobase, and increased the number of action potentials evoked by a depolarizing current at 2X rheobase in neurons from CCD mice. The mechanical and thermal hypernociception after CCD was attenuated by administration of a CXCR4 antagonist AMD3100. These findings suggest that CXCL12/CXCR4 signaling contributes to hypernociception after CCD, and targeting CXCL12/CXCR4 signaling pathway may alleviate neuropathic pain.

## Introduction

Neuropathic pain is one common symptom under various pathological conditions, especially sciatica and low back pain. Pain is normally initiated and mediated by nociceptive primary afferents with their cell bodies in dorsal root ganglia (DRG)^1, 2^. Chronic compression of the dorsal root ganglion (CCD) is a typical model of neuropathic pain, which better mimics low back pain and sciatica in humans^3, 4^. Such pain may accompany an intraforaminal stenosis, a laterally herniated disk, and other disorders that affect the functional properties of the DRG, spinal nerve, or root. Although the pathophysiology of low back pain and sciatica are well studied, the neural mechanisms accompanying pain are not largely explored.

Multiple chemokines have been implicated in neuropathic pain^5–8^. One chemokine, monocyte chemottractant protein-1(MCP-1) was up-regulated by postoperative day 5 in DRG neurons and directly excited injured sensory neurons in compressed L4-L5 DRG in CCD model^7^. Among the chemokines, the chemokine CXC motif ligand 12 (CXCL12), formerly named stromal cell-derived factor 1 (SDF-1) has drawn increasing attention. CXCL12 is typically expressed in stromal cells in various tissues and organs, including skin, thymus, lymph nodes, lung, liver, and bone marrow^9^. In addition, it is also detected in different cell types in the central nervous system (CNS), such as neurons and glias^10^, and the chemokine CXC motif receptor 4 (CXCR4), is a major type of receptor for CXCL12. CXCL12/CXCR4 chemokine signaling has been implicated modulating neuropathic pain associated with the use of nucleoside reverse transcriptase inhibitors (NRTIs) in patients with HIV. The upregulated CXCR4 and CXCL12 expressions in the DRG were involved in nociceptive pain behavior in the rats following ddC (one of the NRTIs) administration^11, 12^. In the rat model of spared nerve injury (SNI), the upregulation of chemokine CXCL12 in the DRG contributed to the development and maintenance of neuropathic pain via the activation of ERK pathway. Moreover, intrathecal injection of AMD3100 reduced the allodynia and levels of p-ERK following SNI^10^. In bone cancer pain model, CXCL12 expression was upregulated in the DRG and spinal cord after tumor cell implantation (TCI) and repeated administration of AMD3100 significantly delayed and suppressed the initiation and persistence of bone cancer pain^13^. The crosstalk between astrocytic CXCL12 and microglial CXCR4 also contributed to the development of neuropathic pain. AMD3100 or minocycline (microglia activation inhibitor), reversed CXCL12-indued mechanical allodynia in naïve mice^14^. These results suggests CXCL12/CXCR4 signaling might participate in development and maintenance of neuropathic pain.

Although CXCL12 is regarded as a key pro-inflammatory mediator in the pathogenesis of neuropathic pain, a possible contribution of CXCL12 and its receptor CXCR4 to neuropathic pain in CCD model has not been examined. Whether CXCL12 and CXCR4 are involved in neuronal hyperexcitability in DRG after CCD remains unknown. We hypothesized that the upregulated CXCL12/CXCR4 signaling directly contribute to the hyperexcitability of DRG neurons after CCD and blocking CXCL12/CXCR4 signaling in DRG may help attenuate mechanical and thermal hypersensitity associated with CCD. We tested this possibility with the use of behavioral testing, PCR, immunofluorescent labeling, calcium imaging, and whole-cell patch clamp recording.

## Results

### Expression of CXCL12/CXCR4 on mouse DRG

**Figure 1:**
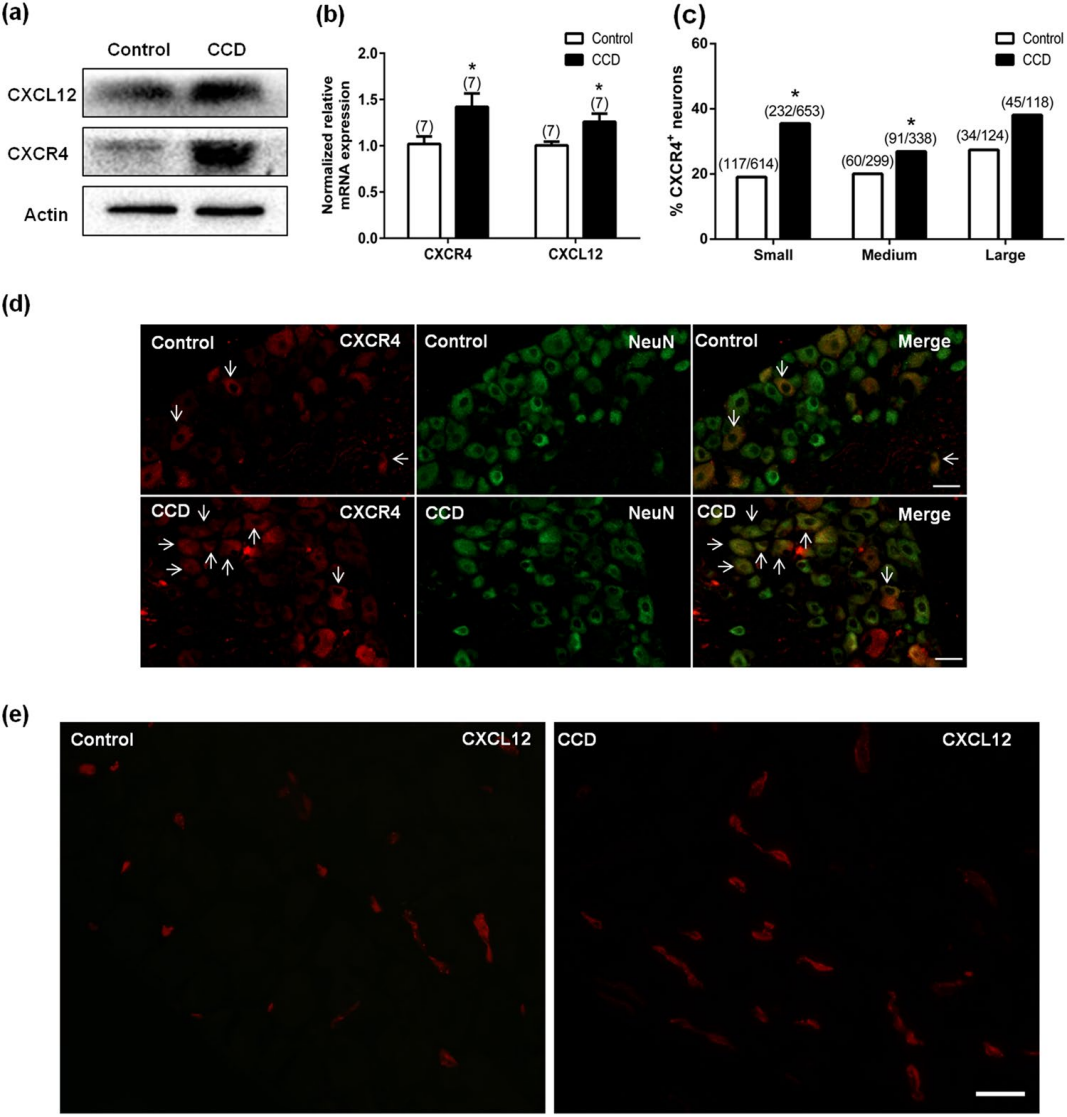
Expression of CXCL12/CXCR4 in mice DRG. (**a**) CXCL12 and CXCR4 protein expressions were increased on DRG after CCD (n = 4). (**b**) Both CXCL12 and CXCR4 mRNA were increased after CCD surgery 7 days (n = 7), *P < 0.05 vs. Control, unpaired t-test. (**c**) The percentages of CXCR4 positive small and medium neurons in the CCD mice were significantly greater than that from control animals, (n = 3, each group). *P < 0.05 vs. Control, Chi-square test. (**d**) Immunoreactivity for CXCR4 was increased after CCD surgery. The arrows indicated some positive neurons. Scale bar: 50 μm. (**e**) Immunoreactivity for CXCL12 from CXCL12^DsRed^ knock-in mice was increased after CCD surgery. Scale bar: 50 μm.

On day 7 after surgery, mRNA and proteins of CXCL12 and CXCR4 were increased in DRG from CCD mice, compared to those from control animals (Fig. 1a,b). We did immunostaining of CXCL12 using CXCL12^DsRed^ knock-in mice. The bright fluorescence produced by DsRed knockin was visualized directly (Supplementary Figure 4). Immunofluorescent staining showed the increased expression of CXCL12 and CXCR4 in DRG (Fig. 1d,e). The percentage of DRG neurons stained with CXCR4 from CCD mice was significantly greater as compared with that from control animals (Fig. 1c), including small- (control:19.06%, 117/614cells ~ CCD:35.53%, 232/653 cells), medium- (control:20.07%, 60/299 cells ~ CCD: 26.92%, 91/338 cells) size neurons. The large-size neurons (control:27.42%, 34/124 cells ~ CCD:38.14%, 45/118 cells) from CCD neurons exhibited a trend of increased CXCR4^+^ percentage, although this did not reach a significant *P* value. However, there were no changes in size distribution of the CXCR4^+^ neurons between control and CCD groups (Supplementary Figure 3).

**Figure 2:**
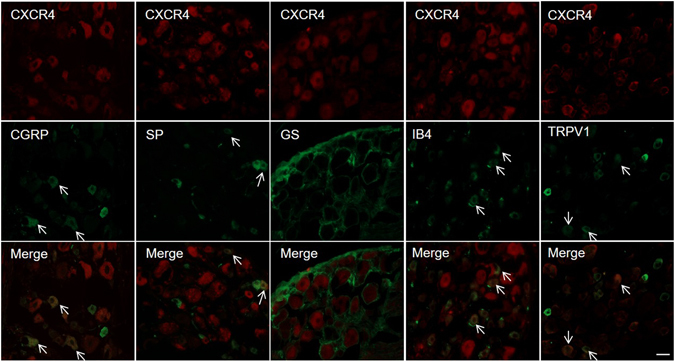
CXCR4 was co-expressed with IB4, SP, TRPV1 and CGRP in DRG neurons (arrows in merged image), but not in the satellite glial cells that were immunopositive for GS from CCD mice on postoperative day 7. Scale bar: 50 μm.

**Figure 3:**
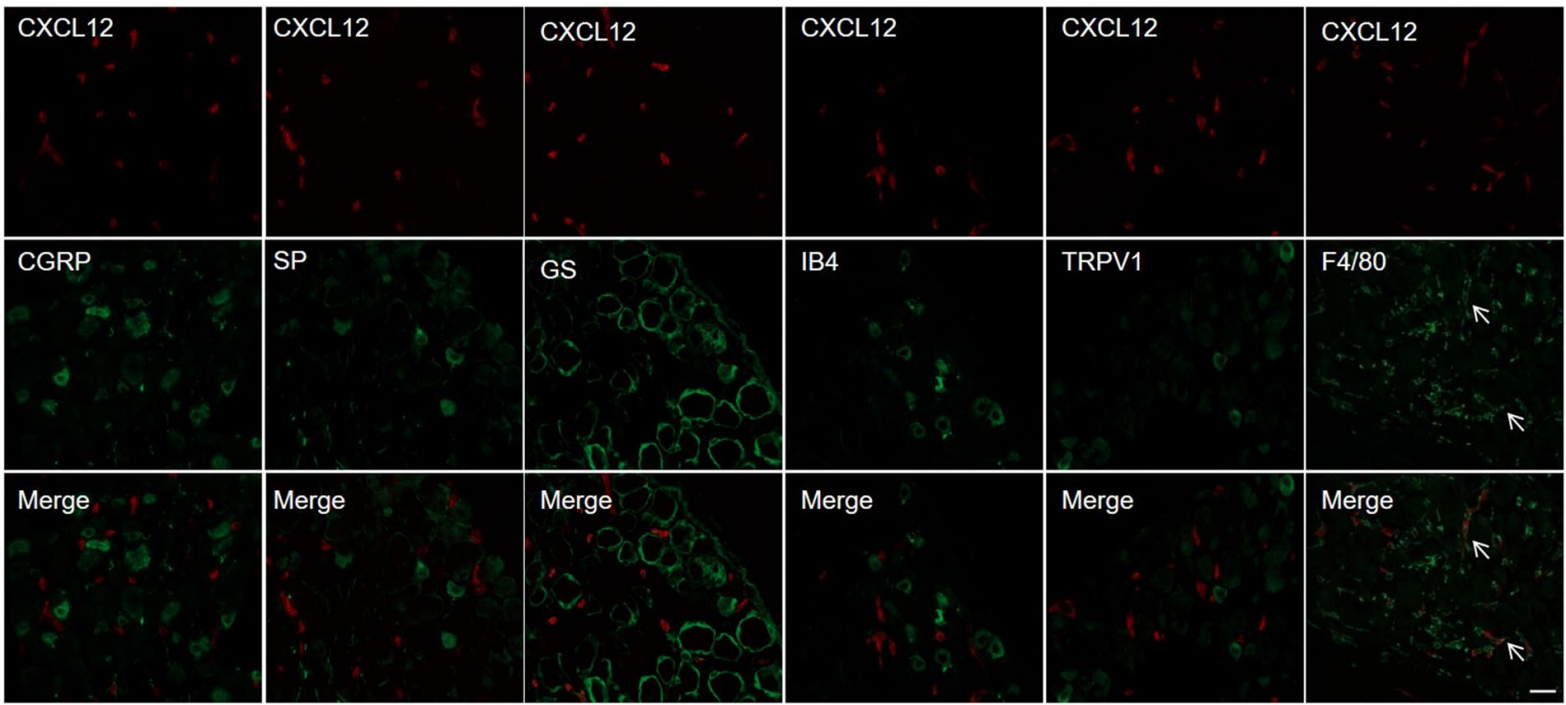
Immunoreactivity for CXCL12 was detected in the macrophages (F4/80) (arrows in merged image), barely co-localized with nociceptive neurons and satellite glial cells from CCD CXCL12^DsRed^ knock-in mice on postoperative day 7. Scale bar: 50 μm.

We further determined the expression pattern of CXCL12/CXCR4 in DRG after CCD. A subset of CXCR4 immunopositive neurons were also immunopositive for the nociceptive neuronal markers IB4, TRPV1,CGRP, and substance P (Fig. 2), but immunoreactivity of CXCR4 was not detected in the satellite glia cells that were immunopositive for GS. Immunoreactivity for CXCL12 from CXCL12^DsRed^ knock-in mice was detected in the macrophages, barely co-localized with nociceptive neurons and satellite glial cells (Fig. 3). In addition, CXCL12 and CXCR4 mRNA expression were not changed in spinal cord at L5 (Supplementary Figure 2).

### CXCL12 induced [Ca^2+^]_i_ increase via neuronal CXCR4 in dissociated DRG neurons

**Figure 4:**
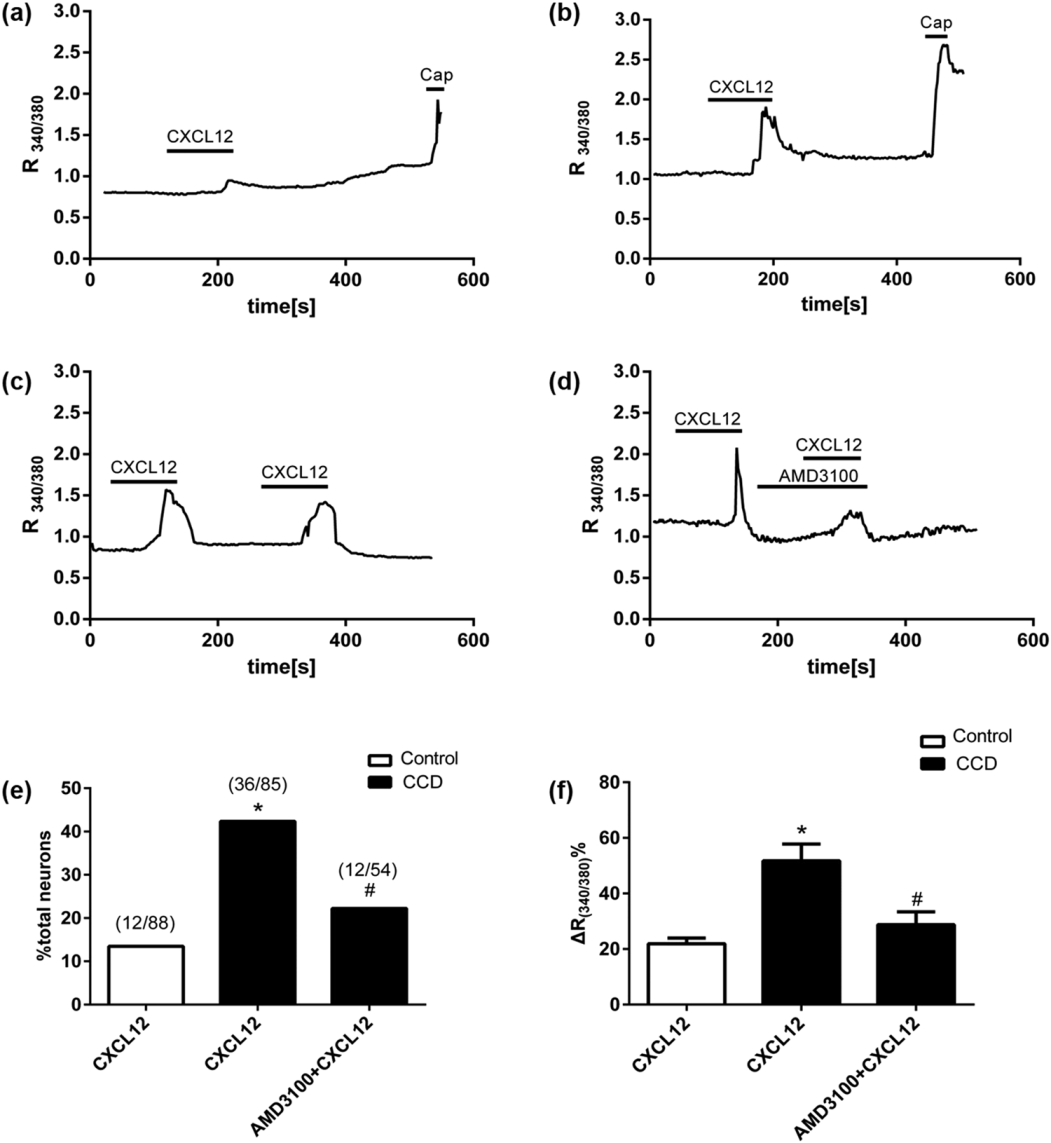
CXCL12 induced [Ca^2+^]_i_ increase through neuronal CXCR4 in the dissociated DRG neurons from CCD mice on postoperative day 7. Black bars above the traces indicate the timing of chemical application. Representative trace showing that CXCL12-induced changes in [Ca^2+^]_i_ (R_(340/380)_) in neurons from CCD mice (**b**) was significantly greater than that in neurons from control mice (**a**). (**c**,**d**) In the presence of AMD3100, the rise in [Ca^2+^]_i_ evoked by CXCL12 was significantly less than that in the control medium without antagonist. (**e**) Quantification of the percentage of DRG neurons that responded to CXCL12, Few (12 of 88 cells, 13.48%) DRG neurons from control mice (n = 6) responded to CXCL12 (100 nM). In contrast, there were more (36 of 85 cells, 42.35%) neurons responed to CXCL12 in CCD mice (n = 8), Also, the percentage of CXCL12 responsive neurons from CCD mice was decreased in the presence of AMD3100 (12 of 54 cells, 22.22%, n = 8). *P < 0.05 vs. (Control + CXCL12) group, ^#^ *P* < 0.05 vs. (CCD + CXCL12) group, Chi-square test. Numbers of neurons tested are given in parentheses. (**f**) Quantification of changes(Δ) in [Ca^2+^]_i_ R_(340/380)_ among responsive neurons. Changes in [Ca^2+^]_i_ R_(340/380)_ was significantly greater for neurons from CCD than from control mice. AMD3100 attenuated CXCL12-induce change in [Ca^2+^]_i_ R_(340/380)_ in neurons form CCD mice, *P < 0.05 vs. (Control + CXCL12) group, ^#^ *P* < 0.05 vs. (CCD + CXCL12), one-way ANOVA followed by Tukey’s post hoc test.

To determine whether the function of CXCL12/CXCR4 signaling in primary sensory neurons was enhanced after CCD, we compared Ca^2+^ responses evoked by CXCL12 in small-diameter DRG neurons between control and CCD mice using ratiometric Ca^2+^ imaging. Few (12 of 88 cells, 13.48%) DRG neurons from control mice (n = 6) responded to CXCL12 (100 nM). In contrast, there were more (36 of 85 cells, 42.35%) neurons responded to CXCL12 in CCD mice (n = 8) (Fig. 4e). Moreover, the amplitude of Ca^2+^ response in neurons from CCD mice was significantly greater than that in neurons from control mice (Fig. 4a,b,f).

To test whether the CXCL12-induced [Ca^2+^]_i_ response was mediated by CXCR4, DRG neurons were treated with a specific CXCR4 antagonist, AMD3100 (5 μM in HEPES buffer), for 2 minutes. In absence of AMD 3100, repetitive application of CXCL12 induced similar Ca^2+^ responses, indicating that no desensitization occurred. In the presence of AMD3100, the rise in [Ca^2+^]_i_ evoked by CXCL12 from CCD mice was significantly attenuated (Fig.4c,d,f). Also, the percentage of CXCL12 responsive neurons from CCD mice was decreased in the presence of AMD3100 (12 of 54 cells, 22.22%) (Fig.4e). AMD3100 alone had no effect on the Ca^2+^ responses in CXCL12 responsive neurons from CCD mice (Supplementary Figure 1).

### CXCR4 activation increased the excitability of DRG neurons

**Figure 5:**
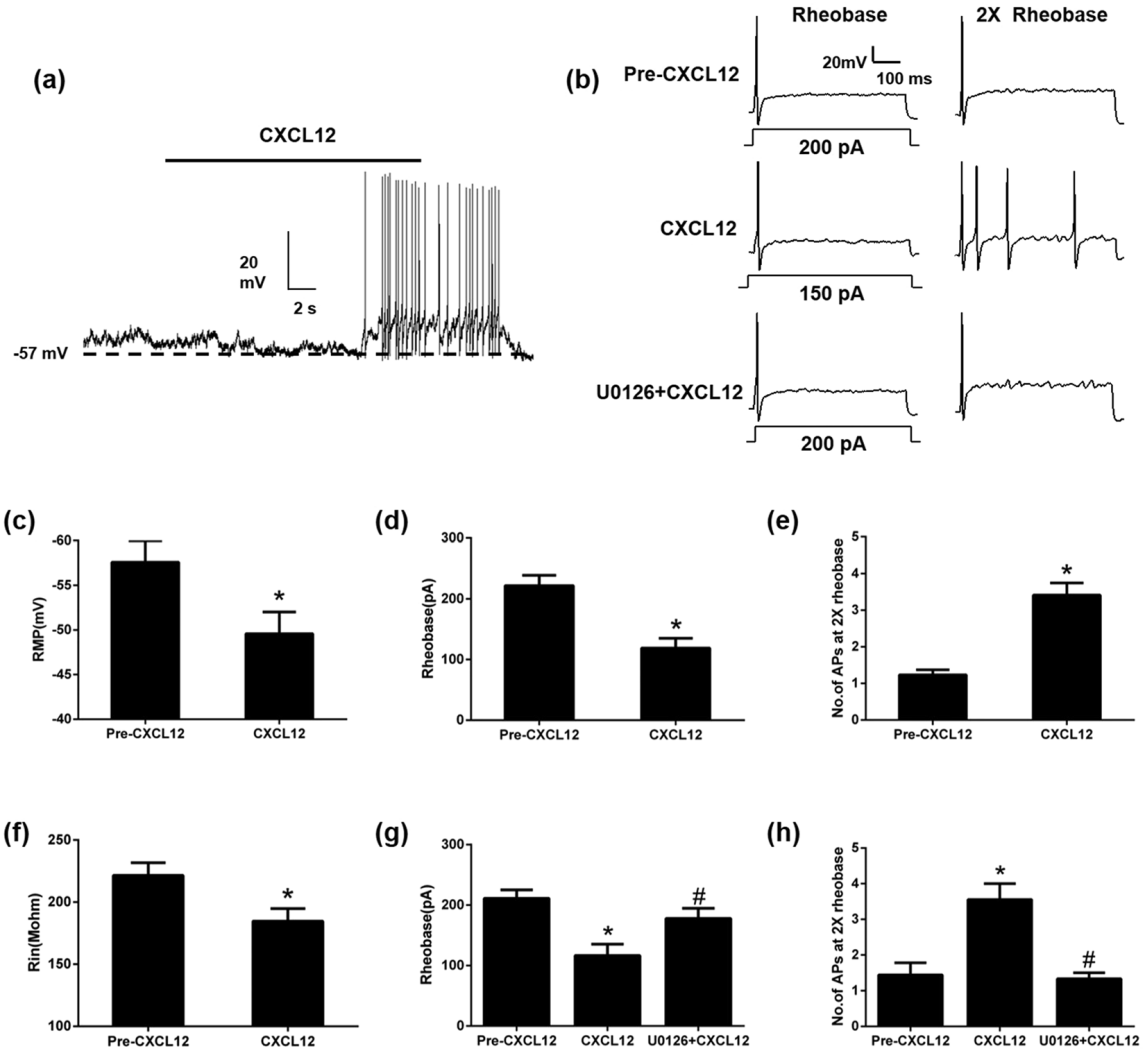
CXCL12 (100 nM) increased the excitability of DRG neurons from CCD mice on postoperative day 7, ERK pathways may be involved in the hyperexcitability of DRG neurons after CCD. (**a**) Typical current-clamp recordings of CXCL12-induced membrane potential depolarization and action potential discharges. (**b**) Typical traces of APs evoked by a 500-ms depolarizing current pulse at 1 and 2 × rheobase before, during CXCL12 application or CXCL12 application with U0126 (20 μM). (**c**) Mean RMP before (Pre-CXCL12) and during CXCL12 application (n = 17), *P < 0.05 vs. Pre-CXCL12, paired t-test. (**d**,**e**) CXCL12 significantly decreased the mean rheobase and increased the number of APs evoked by a depolarizing current at 2 × rheobase (n = 17).*P < 0.05 vs. Pre-CXCL12, paired t-test. (**f**) Mean input resistance (R_in_) before (Pre-CXCL12) and during application of CXCL12 (n = 17), *P < 0.05 vs. Pre-CXCL12, paired t-test. (**g**,**h**) CXCL12 decreased the mean rheobase and increased the number of APs evoked by a depolarizing current at 2 × rheobase, but U0126 treatment inhibited these changes. (n = 10) *P < 0.05 vs. Pre-CXCL12, ^#^ *P* < 0.05 vs. CXCL12, repeated measure one-way ANOVA followed by Tukey’s post hoc test.

In previous study, CCD neurons exhibited a significantly lower rheobase (the minimal depolarizing current required for evoking an AP) than control neurons under current clamp mode^15^. However, little is known whether CXCL12/CXCR4 signaling could alter the excitability of DRG neurons. Bath application of 100 nM CXCL12 depolarized the resting membrane potential (RMP) in DRG neurons of CCD mice, which were identified as responsive to CXCL12 by calcium imaging firstly (Fig. 5c). Furthermore, CXCL12 triggered action potential (AP) discharges in some neurons (Fig. 5a). In addition, bath application of CXCL12 significantly decreased the rheobase (Fig. 5d), and increased the number of APs evoked by a depolarizing current pulse at 2X rheobase (Fig. 5b and e). The input resistance was reduced upon exposure to CXCL12, suggesting an increase in the opening of resting ion channels (Fig. 5f).

To check the involvement of ERK pathway in the CXCL12/CXCR4 mediated hyperexcitability after CCD, U0126, a potent ERK inhibitor was used to check its effect on DRG neuronal excitability during CXCL12 application. Pretreatment with U0126 (20 μM) for 5 min attenuated the excitatory effect of CXCL12 in the DRG neurons from CCD mice (Fig. 5b,g,h).

### Effects of CXCR4 blockade and CXCL12 deficiency on mechanical and thermal hyperalgesia in CCD

**Figure 6:**
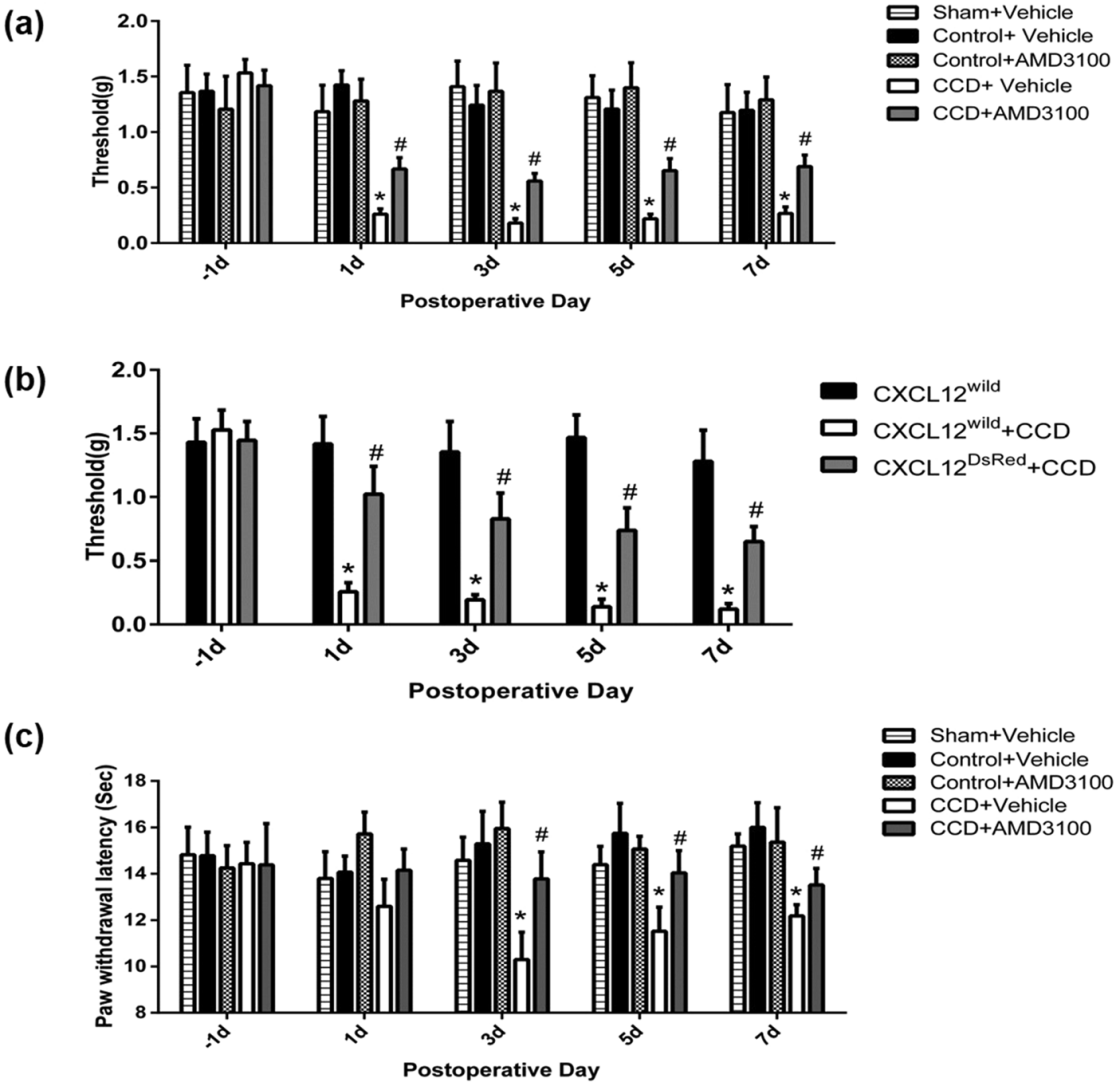
Effects of CXCR4 blockade and CXCL12 deficiency on behavioral postoperative mechanical threshold. Threshold was defined as the force eliciting 50% paw withdrawal. (**a**) The postoperative mechanical thresholds of CCD mice (n = 10) were significantly reduced on postoperative day 1 and remained decreased through day 7, and intraperitoneal injection of AMD3100 ameliorated the tactile allodynia (n = 10) but had no such effects in control mice (n = 6). No obvious mechanical hyperalgesia was observed after sham operation and there were no differences in mechanical threshold between sham (n = 6) and naïve control. *P < 0.05 vs. (control + vehicle) group (n = 10), ^#^ *P* < 0.05 vs. (CCD + vehicle) group, LSD post hoc test following two-way ANOVA with repeated measures. (**b**) After CCD surgery, the CXCL12^DsRed^ knock-in mice (n = 9) with deficient function of CXCL12 showed higher postoperative mechanical thresholds than CXCL12^wild^ + CCD group. *P < 0.05 vs. CXCL12^wild^ group (n = 5), ^#^ *P* < 0.05 vs. (CXCL12^wild^ + CCD) group (n = 7), LSD post hoc test following two-way ANOVA with repeated measures. (**c**) Thermal latencies of CCD mice (n = 7) were significantly reduced on postoperative day 3 and remained decreased through day 7, and intraperitoneal injection of AMD3100 ameliorated the thermal hyperalgesia (n = 8) in CCD mice but not in control mice (n = 6). *P < 0.05 vs. (control + vehicle) group (n = 10), ^#^ *P* < 0.05 vs. (CCD + vehicle) group, LSD post hoc test following two-way ANOVA with repeated measures.

In the ipsilateral hindpaw, the postoperative mechanical threshold of (CCD + Vehicle) mice was significantly decreased compared to pre-CCD values on postoperative day 1 and remained decreased through day 7 (Fig. 6a). To test whether CXCL12/CXCR4 signaling may affect the mechanical allodynia after CCD, AMD3100 (5 mg/kg), a CXCR4 antagonist^16^, was injected intraperitoneally in CCD mice 1 hour before every behavioral test on postoperative day 1, 3, 5, 7. Mechanical hypersensitivity after CCD was partially attenuated by AMD3100 from postoperative day 1 to day 7. AMD3100 (n = 6) had no such effect in control mice (Fig. 6a). To explore the involvements of CXCL12 in neuropathic pain after CCD, the CXCL12^DsRed^ knock-in mice expressing DsRed from the endogenous CXCL12 promoter were used. In these mice, CXCL12 function was impaired. After CCD surgery, postoperative mechanical thresholds from CXCL12^DsRed^ knock-in mice were significantly greater, compared to wildtype CCD animals (Fig. 6b).

Sensitivities of the ipsilateral hindpaws to heat stimuli were tested at the time points of days 1, 3, 5, 7 after operation. Following CCD operation, the thermal latency reflex to radiant heat stimuli was significantly decreased (Fig. 6c). The decrease in thermal latency began on day 3 post operation and persisted through the whole testing period. After AMD3100 administration, postoperative thermal latencies were increased, compared with (CCD + Vehicle) group. Therefore, thermal hyperalgesia after CCD was partially attenuated by AMD3100.

## Discussion

In this study, several evidences have indicated that CXCL12/CXCR4 signaling is enhanced within DRG after CCD.

Although CXCR4 is typically expressed in subsets of immune cells to regulate immunity^17^, CXCL12 and its cognate receptor CXCR4 are constitutively expressed in the DRG and spinal cord in small amount^18, 19^. In present study, RT-PCR revealed an upregulation of CXCL12 and CXCR4 mRNA expression on DRG after CCD. At the protein level, CXCL12 and CXCR4 protein expressions were increased on DRG after CCD. Immunofluorescent labeling indicated that increased number of DRG neurons became CXCR4^+^ after CCD, including small-, medium-sized neurons which also immunopositve for nociceptive neuronal markers, IB4, CGRP, TRPV1 and substance P. Previously studies also showed that CXCR4 and CXCL12 mRNA were induced on postoperative 7 days after spinal cord injury^11, 19^. It is possible that the activation of upstream signaling cascade of cytokines and other mediators may contribute to CXCL12/CXCR4 upregulation. In SNI model, the increased CXCL12 expression in the DRG and spinal cord was inhibited by thalidomide treatment, the inhibitor of TNF-α synthesis, which indicates TNF-α may play a role in this process^10^. In addition, Substance P and CGRP increased in the spinal cord following pSNL model^14^ and induced CXCR4 protein expression^20^, which might explain why CXCR4 was detected from DRG neurons expressing substance P and CGRP. The mechanisms underlying upregulation of CXCL12/CXCR4 signaling in the cell bodies of sensory neurons after CCD need further investigation.

We founded that CXCR4 was mainly present on the somata of DRG neurons, suggesting that CXCL12 might activate the CXCR4 on the DRG neuronal somata. Consistent with previous reports^19, 21, 22^, we found some CXCR4 immunopositive neurons were also immunopositive for IB4, Substance P, TPRV1, CGRP, but CXCR4 immunoreactivity was not found in the satellite glial cells. Reaux-Le and colleagues^21^ have found that CXCR4 receptor is constitutively present in both CGRP and IB4 positive DRG neuronal somata in rats. CXCR4 can be localized in pre-synaptic components of both type I and type II glomeruli in the spinal dorsal horn under electron microscope, indicating that CXCR4 could be axonally transported to both peripheral and central terminals of the primary afferent neurons in DRG and exert its functions^21^. We also found CXCL12 expression was enhanced after CCD and its immunoreactivity was detected in the macrophages, barely co-localized with nociceptive neurons and satellite glia cells. The expression of CXCL12 in the macrophages was also reported in previous studies^9^. However, the CXCL12 was also found both in DRG neurons and satellite cells in other studies^10, 18, 23^. This discrepancy might be due to the different pain models used and the different time points for measurement. The macrophages might be activated by CCD surgery that served as a source of CXCL12 release, allowing its binding to CXCR4 up-regulated in the primary nociceptors, indicating that CXCL12 might activate CXCR4 in a paracrine manner, similar to the condition of neoplastic cells^24^.

We showed that CXCL12 triggered an increase in [Ca^2+^]_i_, after CCD in a subsets of DRG neurons. Consistent with the anatomical observations of CXCL12/CXCR4 expression, our calcium imaging experiments demonstrated a considerable degree of functional CXCR4 upregulation in DRG neurons of CCD mice. A larger proportion of dissociated DRG neurons became responsive to CXCL12 after CCD. The change of incidence was matched to the increased numbers of small-sized CXCR4 positive DRG neurons after CCD. The amplitude of CXCL12-induced Ca^2+^ response in neurons from CCD mice was also significantly greater than that in neurons from control animals. Moreover, Ca^2+^ response was remarkably inhibited by AMD3100. Previously studies reported that CXCL12 dose-dependently increased intracellular calcium in adherent IEC-6 cells and hematopoietic stem and progenitor cells^25, 26^. In diabetic neuropathy model, DRG sensory neurons acutely isolated from diabetic mice displayed enhanced CXCL12 induced calcium responses^27^. The increased [Ca^2+^]_i_ could be used as a readout of neuronal hyperexcitability under neuropathic pain condition^7, 11, 22^. Therefore, these data indicated that CXCL12/CXCR4 signaling might contribute to neuropathic pain in CCD model by directly exciting DRG neurons.

In whole-cell patch clamp experiments, we found CXCL12 enhanced the excitability of DRG neurons through neuronal CXCR4. The underling mechanisms remain to be determined. It is possible that CXCL12/CXCR4 signal activates a Ca^2+^ channel in DRG neurons, leading to hyperexcitability. The Ca^2+^-permeable mechanisms^28, 29^ such as certain TRP channels^30, 31^ might be involved in CXCL12-induced hyperexcitability in DRG neurons. Suppression of functional upregulation of TRPV1 in DRG neurons could reduce hyperexcitability of DRG neurons and pain hypersensitity in bone cancer rats^32^. In our study the co-expression of CXCR4 with TRPV1 supported a possible association of TRPV1 with neuronal hyperexcitability. Additionally, DRG neurons expressed multiple voltage-gated Na^+^ and K^+^ channels that might contribute to the CXCL12-induced neuronal hyperexcitability^23, 33, 34^.

In present study, behavioral test showed that mechanical hypersensitivity and thermal hyperagesia^35^ induced by CCD were attenuated by CXCR4 antagonist, AMD3100. In addition, CXCL12^DsRed^ knock-in mice with deficient function of CXCL12 exhibited less mechanical hyperalgesia after CCD surgery. These results demonstrated that CXCL12/CXCR4 signaling might affect the development of neuropathic pain in the context of nerve injury. ERK^36^ and PI3K pathways may be involved in the pain behaviour^37, 38^. LY294002, a highly selective PI3K inhibitor could prevent CXCL12-induced acute mechanical hyperalgesia at first 24 h after CXCL12 intraplantar injection^37^. CXCL12-induced neuronal hyperexcitability in CCD mice was attenuated by U0126, a potent ERK inhibitor (Fig. 5b,g,h) indicating that ERK may serve as a candidate for downstream signaling pathway of CXCL12. In conclusion, our findings support the hypothesis that CXCL12/CXCR4 signaling was enhanced in DRG after CCD, and that CXCL12 evoked neuropathic pain by directly activating CXCR4 expressed on small-diameter primary sensory neurons. The neuronal mechanism of CXCL12/CXCR4 signaling may play a role in the manifestations accompanying neuropathic pain. Targeting CXCL12/CXCR4 signaling may be helpful in attenuating the clinical radicular pain of patients.

## Methods

### Animals

The experiments were performed on male C57BL/6 mice (20–25 g) from Guangdong Medical Laboratory Animal Center and male CXCL12^DsRed^ knock-in mice (20–25 g) from the Jackson Laboratory (No: 022458, Bar Harbor, ME).The CXCL12^DsRed^ knock-in mice express DsRed protein from the endogenous CXCL12 promoter. A DsRedE2-polyA-Frt-Neo-Frt cassette was inserted into the second exon of CXCL12, produces a strong loss of function phenotype. Animals were housed in groups of 4 or 5 under a 12 h light/dark cycle (with the lights on at 7:00 a.m. to 7:00 p.m.) at room temperature (22–26 °C) with free access to water and food. Animals were randomized to treatment group. The experimental protocols were approved by the Institutional Animal Care and Use Committee of Guangzhou Medical University and were in accordance with guidelines of the National Institutes of Health on the care and ethical treatment of animals.

### CCD surgery

CCD surgery was performed as described previously^15, 33^. In brief, the C57BL/6 mouse was anesthetized with Amobarbital Sodium (50 mg/kg, ip). Intervertebral foramina of lumbar L4–5 were exposed; a stainless L-shaped steel rod, 2 mm in length and 0.3 mm in diameter, was implanted into L4 and L5 intervertebral foramen to compress the L4 and L5 DRGs. The incision was closed in layers. Sham surgeries were performed on 6 mice. The surgical procedure of sham surgery was identical to that described above but without the rod insertion. There were no mechanical hyperagesia after sham operation and there were no difference between sham and naïve control (Fig. 6a). Previous studies also have shown that sham operation have little or no effect on pain behavior or on the electrophysiological properties of cell bodies in the DRG^39, 40^. Then mice received no surgery were used as control groups.

### Behavioral testing

The mice were given normal saline (Vehicle) or AMD3100 (Sigma, St.Louis, MO) 5 mg/kg (dissolved in normal saline) by intraperitoneal injection 1 hour before every behavioral test on postoperative day 1, 3, 5, 7 for different groups. Mice were placed in a transparent glass chamber (9.5 × 7.0 × 4.5 cm) placed over a metal mesh wire mounted on a raised metal platform. To habituate the mice to the experimental conditions, the mice were handled and placed in the experimental chambers, and their hindpaw were periodically stimulated by randomly selected Touch Test Sensory Evaluator Evaluator (North Coast Medical, Inc, USA) once a day for 1 week before the onset of behavioral data collection and subsequent CCD surgery. Mechanical hyperalgesia was measured in the hindpaw before and after using the up-down method. The experimenters were blinded to the treatments and mouse genotypes^18, 41^.

To examine thermal hyperalgesia, the mouse was placed on the surface of a 5-mm thick glass covered with a Plexiglas box. The sensitivity to heat stimuli was measured with a radiant heat stimulator (IITC Inc, CA). The heat source produced a 4 × 6 mm light spot, which was directed onto the plantar surfaces of the hindpaws, 10-min intervals in the same paw. Five stimuli were administered at the same testing site. The latency of paw withdrawal was recorded. The test was conducted before operation and on days 1, 3, 5, 7 after CCD operation. To avoid excessive tissue injury, the maximal duration of heat stimulation was 30s^35^. The mice were given normal saline (Vehicle) or AMD3100 (Sigma, St.Louis, MO) 5 mg/kg (dissolved in normal saline) by intraperitoneal injection 1 hour before every behavioral test on postoperative day 1, 3, 5, 7 for different groups.

### PCR

Real-Time PCR was used to assess CXCR4 and CXCL12 mRNA regulation after CCD. L4 and L5 lumbar DRG were dissected in control mice and CCD mice. Total RNAs of DRG were extracted using the RNeasy Plus Micro Kit (Qiagen, Hannover GmbH, Germany) according to the manufacturer’s protocol. 0.3 μg of total RNA was reversely transcribed to cDNA using maxima H minus First strand cDNA synthesis kit (Thermo Scientific, Rockford, IL), according to the manufacturer’s instructions. Real-time quantitative PCR was performed with the above prepared cDNA and SYBR Green Master Mix (Invitrogen, Carlsbad, CA). The primers for CXCR4, CXCL12 and β-actin were as follows: Forward Primer (CXCR4) 5′-AGGAAACTGCTGGCTGAAAAGG-3′, Reverse primer (CXCR4) 5′-GGAATTGAAACACCACCATCCA-3′; Forward Primer (CXCL12) 5′-GTCTAAGCAGCGATGGGTTC-3′, Reverse primer (CXCL12) 5′-GAATAAGAAAGCACACGCTGC-3′; Forward Primer (β-actin) 5′-GCATTGCTGACAGGATGCAG-3′, Reverse primer (β-actin) 5′-CCTGCTTGCTGATCCACATC-3′. The amplification conditions were 10 min at 95 °C, followed by 40 cycles of 10 s at 95 °C, 30 s at 60 °C and extension at 72 °C for 30 sec. Quantitation of mRNA was performed by using Applied Biosystem ViiATM 7 Real-time PCR System (Applied Biosystem, Foster city, CA). The gene β-actin was used to normalize the mRNA levels of each sample.

### Immunohistochemistry

Immunofluorescent labeling of the following markers was performed on control and CCD mouse lumbar DRG cryosections using the methods as previously described^7^: NeuN (neuronal marker), CXCR4, glutamine synthetase (GS, as a marker for satellite glial cells^42, 43^), isolectin B4 (IB4), transient receptor potential vanilloid 1 (TRPV1), substance P (SP), F4/80 (the macrophage marker) and calcitonin gene-related peptide (CGRP). CXCL12, the bright fluorescence produced by DsRed knockin can be detected directly^44^. The staining pattern of CXCL12 was identical using anti-RFP antibody or not (Supplementary Figure 4). Briefly, mice were transcardially perfused with saline solution followed by 4% paraformaldehyde, the L4 and L5 DRGs were harvested post-fixed in the same fixative for overnight, and then dehydrated in 30% sucrose. The tissue was frozen and sliced at 5 μm thickness in the Cryostat Microtome (CM1950, Leica). Tissue slices were incubated with blocking buffer (3% BSA and 0.2% Triton X-100 in PBS) for 1 h, followed by overnight incubation with the primary antibodies (goat-anti-CXCR4, rabbit-anti-CXCR4, 1:500, Abcam; mouse-anti-GS, 1:100, Abcam; mouse-anti-TRPV1, 1:100, Abcam. rabbit-anti-TRPV1, 1:2000, Neuromics; mouse-anti-SP, 1:200, Abcam; goat-anti-CGRP, 1:1000, Abcam; mouse-anti-F4/80, 1:100, Abcam; chicken-anti-NeuN, 1:200, Abcam) at 4 °C overnight, and then with the proper fluorescence secondary antibodies (donkey-anti-goat, 1:1000; donkey-anti-rabbit, 1:1000, goat-anti-mouse,1:1000, Invitrogen) 37 °C for 1 h. FITC-conjugated IB4 (20 μg/ml, Sigma–Aldrich) was added with the secondary antibodies. The slides were then washed in PBS and cover-slipped with ProLong Gold antifade reagent (Invitrogen, Carlsbad, CA). The cells were visualized and the images were captured using a laser microscopic imaging system (LMS 510, Carl Zeiss MicroImaging). DRG neurons were classified according to their cross-sectional areas as small- (area < 600 μm^2^), medium- (area 600 ~ 1200 μm^2^) and large-sized (area > 1200 μm^2^)^7^. To quantify the immunofluorescence staining in the DRG, the numbers of CXCR4-positive cells per section were counted. In each mouse, 3 ~ 4 sections of the L4–5 DRG at different groups were selected randomly. The percentages of CXCR4-postive cells relative to the total number of cells were obtained for animals across the different sections.

### Western bolt

The DRG of CCD mice (n = 4) were combined as one sample for western blot, the DRG of control mice (n = 4) were combined as the same way. Briefly, L4 and L5 lumbar DRG were dissected in control mice and CCD mice and placed temporarily in liquid nitrogen. Then the samples were homogenized in ice-cold lysis buffer by ultrasonic homogenizer (Cole parmer instruments, USA). The crude homogenates were centrifuged at 4 °C for 15 min at 3 000 rpm, and the supernatants were collected. After the protein concentrations were determined, the samples were heated for 5 min at 99 °C, and 30–60 μg protein was loaded onto 12% SDS–polyacrylamide gels, then electrophoretically transferred onto PVDF membranes. The membranes were blocked with 3% non-fat milk for 1 h and incubated overnight at 4 °C with primary antibody. The following primary antibodies were used: rabbit anti-CXCL12 (1:200, Abcam), rabbit anti-CXCR4 (1:200, Abcam), and mouse anti-β-actin (1:1000, CST). The proteins were detected with horseradish peroxidase-conjugated anti-rabbit secondary antibodies (1:1000, CST), visualized using the supersignal west pico chemiluminescence substrate (Thermo. USA), and exposed in Bio-rad chemiDox-XRS imagine system.

### Cell Culture

Cell culture was prepared as described previously^45^. In brief, at postoperative days 5–7, control mice or CCD mice were anesthetized with Amobarbital Sodium (50 mg/kg ip), and the L4 and L5 DRGs were dissected out. The DRGs were placed in cold, oxygenated Complete Saline Solution (CSS), consisting of (in mM) 137 NaCl, 5.3 KCl, 1 MgCl_2_, 3 CaCl_2_, 25 Sorbitol and 10 HEPES (pH 7.2). For 20 min, the DRGs were digested with 0.35U/ml of Liberase TM (Roche, Manheim, Germany), then for 15 min with 0.25U/ml Librease TL (Roche, Manheim, Germany) and 30U/ml papain (Sigma, USA) in CSS containing 0.5 mM EDTA at 37 °C. The DRG neurons were suspended in DMEM medium containing 1 mg/mL trypsin inhibitor (Roche, Manheim, Germany) and 1 mg/mL bovine serum albumin (Sigma, USA) and then plated onto poly-D-lysine/laminin-coated glass coverslips (Bio-Coat; BD Biosciences, San Jose, CA). The DMEM medium had equivalent amounts of DMEM and F12 (Gibco, Grand Island, NK) with 10% FCS (Gibco, Auckland, New Zealand) and 1% penicillin and streptomycin (Invitrogen, Grand Island, NK). The cells were maintained in 5% CO_2_ at 37 °C in a humidified incubator and used within 16–24 hours after plating.

### Calcium imaging

The selective fluorescent probe, Fura 2-acetoxymethyl ester (5 μM, Dojindo, Japen), was used to measure [Ca^2+^]_*i*_. The cultured mouse DRG neurons were loaded Fura 2/AM in the dark for 30 min at 37 °C. After loading, DRG neurons were washed twice in HEPES buffer to remove extracellular dye, and placed in a recording chamber continuously perfused with HEPES buffer at a flow rate of 1.5 ml/min at room temperature. The HEPES buffer contained (in mM): 145 NaCl, 3 KCl, 2 MgCl_2_, 2 CaCl_2_, 10 glucose and 10 HEPES (adjusted to pH 7.4 with NaOH). Ratiometric calcium imaging was performed at room temperature (20–22 °C) using an upright Olympus BX-51WI microscope equipped with a ratiometric imaging system. The calcium signals by 340 and 380 nm excitationwere recorded at 2-s intervals using a sCMOS camera (PCO, Germany) controlled by a computer with MetaFluor software (Molecular Devices, Sunnyvale, CA). The ratio of 340 nm/380 nm fluorescence intensity (R340/380) within a certain region of interest after background subtraction was used as a relative measure of intracellular calcium concentration ([Ca^2+^]_i_). Therefore, only small-diameter neurons (<30 μm) with R340/380 at the range of 0.7–1.25 were included in this study. Neurons were considered capsaicin sensitive (CAP^+^) if a 10-s application of 1 μM capsaicin evoked an increase in R340/380 that was equal or greater than 15% above baseline. The proportion of DRG neurons responsive to CXCL12 (15% above baseline) was calculated as the number of CXCL12-responsive neurons through a micropipette and a 6-channel drug application system (VC-6, Warner Instruments, Hamden, CT).

### Electrophysiological recording

Whole-cell patch-clamp recordings were performed on dissociated CCD DRG neurons at room temperature using a Multiclamp 700 A amplifier with Pclamp 10.5 software (Molecular Device, Sunnyvale, CA) as described^6, 46, 47^. Patch pipettes were pulled from borosilicate glass capillaries (Sutter Instrument; 1.5 mm outer diameter, 0.86 mm inner diameter; Novato, CA) using a horizontal puller (Model P97, Sutter Instrument, Novato, CA). The resistance of the patch pipettes was 3–4 M when filled with an internal solution consisting of (in mM): K^+^-gluconate 120, KCl 20, CaCl_2_ 1, MgCl_2_ 2, EGTA 11, HEPES-K^+^ 10, MgATP 2, adjusted to a pH of 7.2 with Tris-base and having an osmolarity of 290–300 mOsm^46, 47^. Resting membrane potential (RMP) was recorded for each neuron under the current clamp mode after stabilization (within 4 min). A neuron was included if the RMP was more negative than −40 mV and the spike overshoot was >15 mV. Action potentials (APs) were evoked by a series of depolarizing current steps, each 500 ms duration, in increments of 50 pA up to 1 nA. The rheobasewas defined as the minimal depolarizing current required evoking an AP. The number of APs evoked by a suprathreshold stimulus was estimated by injecting a 500-ms depolarizing current of a magnitude at twice the rheobase. Input resistance was obtained from the slope of a steady-state current-voltage plot in response to a series of hyperpolarizing currents steps from −200 to −50 pA.

### Statistical analysis

Data values were presented as mean ± SEM. A Student’s t-test was used to test the statistical significance of a difference between mean responses for two groups. Statistical comparisons of differences among three or more groups were made with a one-way analysis of variance followed by Tukey’s post hoc test. The changes of behavioral testing over time among groups were tested using two-way ANOVA with repeated measures, followed by LSD post hoc test. Chi-Square tests were used to compare the incidence of neuronal responses. The criterion for statistical significance was a value of *P* < 0.05.

## Electronic supplementary material

Supplementary Information 
