# Supplementary material for: Effect of CXCL12/CXCR4 signaling on neuropathic pain after chronic compression of dorsal root ganglion
Source: Sci Rep. 2017 Jul 18;7:5707. doi: 10.1038/s41598-017-05954-1 (PMC5515923; doi:10.1038/s41598-017-05954-1)
Supplement: Supplementary file 1 — Supplementary Information [file 41598_2017_5954_MOESM1_ESM.doc]

*SUPPORTING INFORMATION FOR*

**Effect of CXCL12/CXCR4 signaling on neuropathic** **pain after chronic compression of dorsal root ganglion**

Yang Yu^a†^, Xini Huang^a†^, Yuwei Di^b^, lintao Qu^c^, Ni Fan^a*^





**Supplementary Figure 1** Representative trace showing that in neurons from CCD mice which responded to CXCL12, application of AMD3100 along has no effect to the [Ca^2+^]_i_.





**Supplementary Figure 2** Expression of CXCL12/CXCR4 in spinal cord at L5. Both CXCL12 and CXCR4 mRNA weren’t increased in spinal cord after CCD surgery 7 days (n=3), unpaired t-test.

**Method:** In brief, at postoperative days 5–7, control mice or CCD mice were anesthetized with Amobarbital Sodium (50 mg/kg ip), and the L5 spinal cords were harvested. Total RNAs of spinal dorsal horns were extracted using the RNeasy Plus Micro Kit (Qiagen, Hannover GmbH, Germany) according to the manufacturer’s protocol. The protocol and primers were the same as PCR from **Methods of the article.**





**Supplementary Figure 3** The size distribution of the CXCR4^+^ DRG neurons from control (n=3) and CCD group (n=3). There weren’t significant statistical differences in size distribution of CXCR4^+^ neurons between control and CCD groups, Chi-square test.


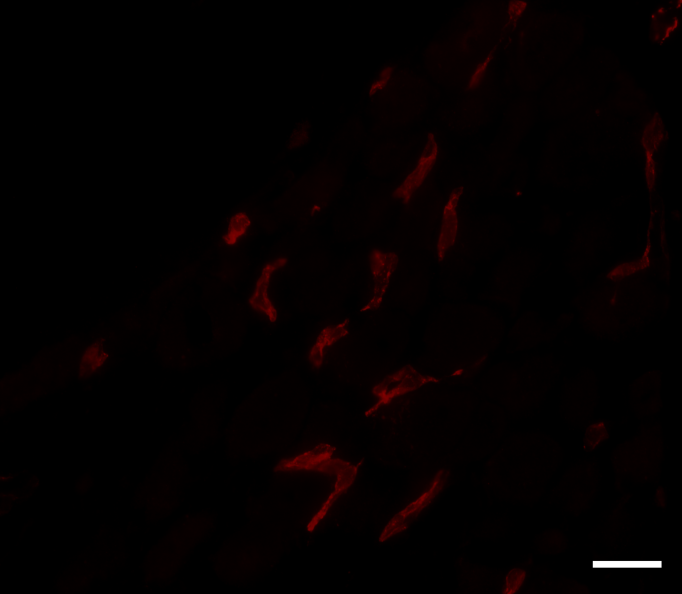

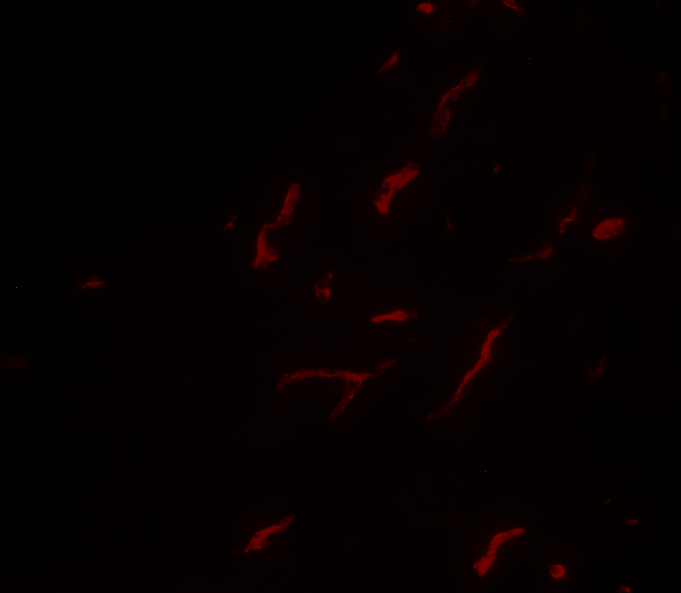


CXCL12*^DsRed^*

CXCL12*^DsRed^*

**Supplementary Figure 4** The staining pattern of CXCL12 was identical using anti-RFP antibody or not. Left: The bright fluorescence of CXCL-12 can be detected directly in CXCL12^DsRed^ knock-in mice. Right: The staining of CXCL12 using anti-RFP antibody. Scale bar: 50 μm.
